# Quality, availability and storage conditions of oxytocin and misoprostol in Malawi

**DOI:** 10.1186/s12884-020-2810-9

**Published:** 2020-03-29

**Authors:** Nhomsai Hagen, Felix Khuluza, Lutz Heide

**Affiliations:** 1 Pharmaceutical Institute, Eberhard Karls University Tuebingen, Tuebingen, Germany; 2 Pharmacy Department, College of Medicine, University of Malawi, Blantyre, Malawi

**Keywords:** Oxytocin, Misoprostol, Post-partum haemorrhage, Medicine quality, Substandard medicine, Falsified medicine, Medicine storage, Medicine analysis, Rational use, Malawi

## Abstract

**Background:**

Postpartum haemorrhage (PPH) is the leading cause of maternal mortality in low- and middle-income countries (LMICs). Oxytocin and misoprostol are used for the prevention and treatment of PPH. However, both medicines are chemically unstable and sensitive to environmental conditions. Previous studies reported a high prevalence of substandard oxytocin and misoprostol preparations in LMICs.

**Methods:**

In randomly selected health facilities of four districts of Malawi, the availability of oxytocin and misoprostol was determined, and the knowledge of health workers on storage requirements and use of oxytocics was assessed. Temperature loggers were used to record the storage temperature of oxytocics. Samples of oxytocin injections and misoprostol tablets were collected from the health facilities and from wholesalers. Oxytocin samples were analysed for identity, assay (= quantity of oxytocin) and for pH value according to United States Pharmacopeia 40. Misoprostol samples were analysed for identity, assay, dissolution and related substances according to the International Pharmacopeia 2017.

**Results:**

All visited hospitals and health centers had oxytocin available. At non-refrigerated storage sites, the recorded mean kinetic temperature exceeded the oxytocic’s storage temperature stated on the labels in 42% of the sites. At refrigerated storage sites, the required temperature of 2–8 °C was exceeded in 33% of the sites. Out of 65 oxytocin samples, 7 (11%) showed moderate deviations from specification, containing 82.2–86.8% of the declared amount of oxytocin. Out of 30 misoprostol samples, 5 (17%) showed extreme deviations, containing only 12.7–30.2% of the declared amount. The extremely substandard misoprostol was reported to the national authorities and to WHO, leading to an immediate recall of the respective brand in Malawi. The UK-based distributor of this brand closed its business shortly thereafter.

**Conclusion:**

Availability of oxytocin was excellent in Malawi, and its quality was better than reported in previous studies in other LMICs. However, storage conditions at the health facilities often did not meet the requirements. Extremely substandard misoprostol tablets were found, representing a serious risk to maternal health. This shows the need for continued efforts for quality assurance in medicine procurement and registration, as well as for post-marketing surveillance.

## Background

**Fig. 1:**
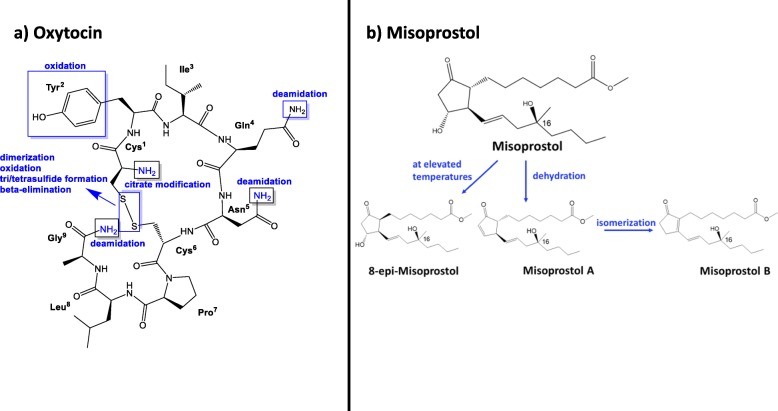
Structures of oxytocin (modified from [2]) and of misoprostol, and their typical degradation mechanisms. Commercial misoprostol is a mixture of the depicted structure at its epimer at C_16_, and of the enantiomers of both compounds. Corresponding stereoisomers are found for the degradation products of misoprostol

Post-partum haemorrhage (PPH) is the leading cause of maternal mortality in low-income countries. It is defined as blood loss of 500 ml or more within 24 h after birth [1]. The most common cause of PPH is an atonic uterus. This condition can be prevented and treated with oxytocics like the nonapeptide oxytocin or the prostaglandin analogue misoprostol (Fig. 1). Their mode of action is uterine contraction [1]. Both oxytocin and misoprostol are listed among 13 life-saving commodities for women and children by the UN Commission on Life-Saving Commodities for Women and Children (UNCoLSC) [3]. For oxytocin and misoprostol, it was estimated that 15,000 maternal lives could be saved over 5 years if common barriers like poor quality of these medicines, or lack of their inclusion into national essential medicines lists, could be overcome and” equitable access” could be achieved, according to the commissioners´ report of 2012 [4].

Reducing global maternal mortality to less than 70 per 100,000 live births is one of the Sustainable Development Goals (SDGs) of the United Nations (UN) [5]. There is still a long way to go to achieve this goal, especially in low-and-middle income countries (LMICs): The maternal mortality ratio in Malawi is one of the highest in the world, with an estimated 439 maternal deaths per 100,000 live births according to the Malawi Demographic and Health Survey 2015–16 [6]. One important intervention to reduce the maternal mortality ratio is the prevention and treatment of PPH with oxytocics. In the fifth edition of the Malawian Standard Treatment Guidelines (MSTG) including the Malawi Essential Medicines List (MEML) of 2015, oxytocin and misoprostol are listed as “vital” oxytocics to treat PPH [7]. Unfortunately, these medicines are very sensitive to environmental conditions like high temperatures (oxytocin, [8–10]) and humidity (misoprostol, [11, 12]). Most oxytocin products should be stored at 2–8 °C according to the labelling, and this can be challenging, especially in rural areas in LMICs [13–16]. Since misoprostol tablets can be stored at room temperature and can be administered orally, they offer an alternative to oxytocin injections in places where appropriate storage conditions for oxytocin cannot be ensured, or where no trained staff is available to administer medicines parenterally [17]. However, misoprostol tablets degrade when exposed to humidity (Fig. 1) and must be packed in aluminium-aluminium blisters in order to avoid degradation [11, 12, 18]. Misoprostol is also used in gynaecology and obstetrics for induction of labour and for abortions [17]. Due to the latter, its use is often restricted for fear of misuse.

The degradation products known for oxytocin or misoprostol are inactive but not toxic [12, 19]. However, when oxytocics lose their potency e.g. due to inappropriate storage, this might result in higher mortality rates of PPH.

Several previous studies have shown that the quality of oxytocics, especially in low and middle-income countries (LMIC), is often poor [8, 11, 15, 16, 20–22]. According to a review from 2016, which included 8 studies with 559 oxytocin samples from 15 countries, 57.5% of the samples collected in Africa were reported to be substandard [20]. Similarly, 45% out of 215 misoprostol samples collected in 15 low- and middle-income countries were substandard according to a paper published in WHO Drug Information in 2016 [11]. A recently published study conducted in Nigeria reported even 74.2% of analysed oxytocin injections to be out of specification, as well as 33.7% of analysed misoprostol tablets [16]. Poor quality of oxytocin vials and misoprostol tablets can result from poor manufacturing (e.g. inappropriate formulation, environmental conditions, or poor primary packaging), from poor storage and transportation conditions, or from a combination of these factors.

But not only availability and good quality of oxytocics are essential for lowering maternal mortality; health workers´ knowledge of rational use of oxytocics, e.g. when and how to administer oxytocics, is as important. According to the World Health Organisation (WHO), one of the key interventions to promote rational use is the use of clinical guidelines [23].

A paper including data on the availability of oxytocin and misoprostol in Malawi has recently been published [24]. However, so far there are no data in the scientific literature about the quality of oxytocin and misoprostol in Malawi, and neither on the storage conditions of these medicines, or on health workers’ knowledge of storage requirements and of rational use of oxytocics.

The present study aimed to close this gap by collecting oxytocin and misoprostol samples at different points of the supply chain and in different health facilities in four districts of Malawi, and by investigating their quality according to the acceptance criteria of the United States Pharmacopeia and the International Pharmacopeia. Furthermore, availability and storage conditions of oxytocics were investigated, and health workers’ knowledge of storage requirements and rational use of these medicines was examined by using a questionnaire (see below).

## Methods

The study protocol was developed based on MEDQUARG guidelines [25] and on the WHO guidelines on the conduct of surveys of the quality of medicines [26].

### Ethical approval

Ethical clearance to conduct this study has been granted by College of Medicine Research and Ethics Committee in Malawi (COMREC, Reference No. P.07/27/2215). We also obtained support letters from the Malawian medicine regulatory authority (Pharmacy, Medicines and Poisons Board, PMPB), as well as from the responsible district health officers. Additionally, approval by German authorities to import medicine samples for analysis was obtained.

Written informed consent to participate in this study was obtained from all interviewed persons (see Consent Form in Additional File 2).

### Sample size calculation

The sample size was calculated using the Cochran formula n_0_ = Z^2^pq/e^2^ [27] with Z = 1.96 at 95% confidence level, *p* = 57.5% estimated proportion of substandard oxytocin samples [20], q = 1-p, and e = 10% margin of error. This resulted in a minimum of 94 samples.

### Selection of sampling sites

This study was conducted in four districts in central and southern Malawi: Blantyre, Chikwawa, Neno and Ntcheu. Blantyre represents an urban district with moderate climate, Ntcheu a rural district with moderate climate, Chikwawa a rural district with hot climate, and Neno a rural district which has areas with both hot and moderate climate. A list of all health facilities in these four districts was obtained from the government-operated “Central Medical Stores Trusts” (CMST) and from PMPB. This list included 4 district or central hospitals, 61 public health centers, 28 faith-based health centers, 103 private clinics (most of them in Blantyre), 26 licensed pharmacies (all of them in Blantyre) and 149 drug stores (most of them in Blantyre). For the present study, the district or central hospital of each district was selected. Furthermore, for each district two facilities of each of the other five types were selected randomly if available, using the RAND function of Microsoft Excel. However, the very unequal distribution of facilities in the four districts required an adjustment of the numbers of selected facilities: licensed pharmacies existed only in Blantyre district, and therefore eight pharmacies (and no drug stores) were randomly selected in this district, plus public and faith-based health centers, private clinics and the central hospital. In Chikwawa and Ntcheu districts, the missing pharmacies, drug stores and private clinics were compensated for by inclusion of more public and faith-based health centers, aiming at a total number of 11 health facilities for each of these two districts. For the small Neno district, only 8 facilities in total were listed by CMST and PMPB, therefor all of these were included.

In the course of the study visits, eight of the selected facilities were found to be out of operation, did not offer maternity services, or refused to collaborate. These facilities were excluded from this study and were replaced by the geographically nearest facility of the same type, and if that facility could not be included either, by the second nearest one. If both attempts were unsuccessful, no further attempt was made to replace that facility. Also if facilities were found to be out of stock for oxytocics, sampling was attempted in the geographically nearest facility of the same type, and if necessary in the second nearest one, as described above.

In addition to health facilities, oxytocics were also requested from CMST and from the 12 most important pharmaceutical wholesalers in Malawi; five of these reported that they did not stock oxytocics.

**Fig. 2:**
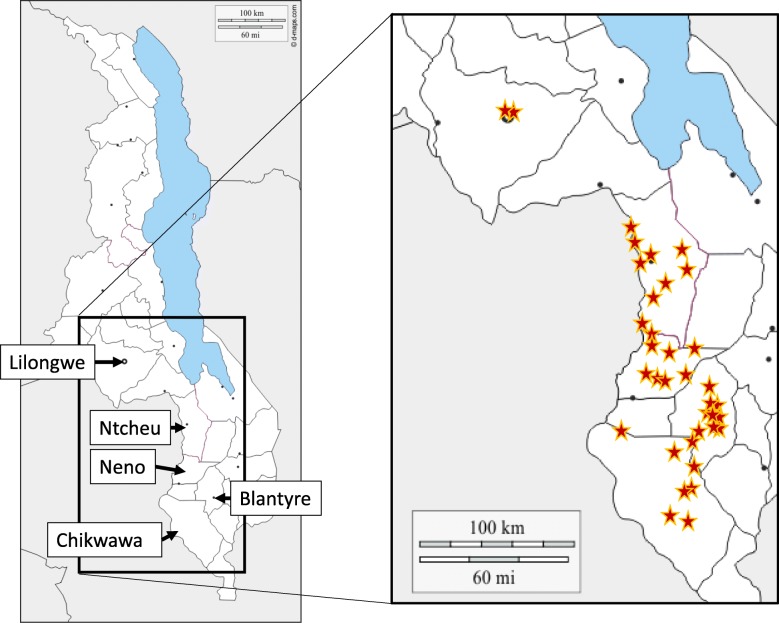
Map of the locations of the 45 sites from which oxytocics were collected. Medicines were collected from the indicated four districts, and from wholesalers in Blantyre city and in Malawi’s capital Lilongwe. Modified from: https://d-maps.com/carte.php?num_car=24744&lang=en

In total, oxytocics were requested from 62 health facilities and drug outlets, and these are listed in Additional File 1. Oxytocics could be collected from 45 of these facilities, and a map with the location of these 45 sampling sites is shown in Fig. 2. At most health facilities, oxytocics were stored both at the pharmacy (storage room) and at the maternity ward, and in each facility, sampling was done from both sites when possible, as listed in Additional File 1.

### Collection of samples

Sampling was conducted between September 2017 (pilot study in Ntcheu district) and August 2018 (Blantyre, Chikwawa and Neno districts). All facilities in one district were sampled within 1 week. Permission to sample was obtained from the responsible district health officer prior to sampling, but facilities were visited without prior notice to the respective facility to avoid bias which might have arisen if the site prepared for our visit.

Sample collectors were the investigators F.K. and N.H., and 3rd year pharmacy students of the College of Medicine, University of Malawi (Blantyre) who had been trained prior to sampling. In hospitals, health centers and private pharmacies, sampling was done by an overt approach, i.e. facilities were informed about the purpose of the visit, and consent to participate in the study was obtained (see Additional file 2). In these facilities, temperature loggers were placed, and interviews were conducted using a questionnaire (see below). In the case of wholesalers and drug stores, a mystery shopper approach was used, and neither placement of temperature loggers nor interviews were carried out.

If different brands (original, generic or branded) or batches of oxytocics were available at a sampling site, each brand and each batch was collected as a separate sample. For each sample, 10 vials of oxytocin and 50 tablets of misoprostol were collected if possible. If only a smaller amount was available, this smaller amount was collected, but not less than three vials of oxytocin or six tablets of misoprostol per sample. Additional File 1 lists the number of samples, and the size of these samples, collected from each sampling site.

In health facilities, replacements for the sampled medicines were offered by the sample collectors in order to avoid that stock-outs would result from this study. The replacement medicines were obtained from CMST and local wholesalers prior to the sampling visits. If the visited facilities preferred, the sampled medicines were paid for, and this was obviously the rule in case of private pharmacies.

### Questionnaire

A short interview on the health worker’s knowledge about storage requirements, and about rational use of oxytocin and misoprostol, was conducted at all health facilities, as well as in those private pharmacies which stocked oxytocics. The questionnaire used for these interviews is shown in Additional File 2. It was written in English which is the language used in the training of all health workers in Malawi. However, the native sample collectors were free to use the local language (Chichewa), and this was the language used in most of the interviews. The sample collectors filled the questionnaire on-site based on the responses of the health workers. As shown in Additional File 1, the questionnaire was applied in 31 health facilities (hospitals, health centers and private clinics) as well as in 6 private pharmacies. In 24 of the 31 health facilities, the interview was carried out both in the storage room (pharmacy) and in the maternity ward, with the person responsible for storage or for administration of oxytocics at the respective site. In four further health facilities, the interview was carried out only at the maternity ward, and in three facilities, only at the storage room. The interview was therefore conducted with personnel from 28 maternity wards, 27 storage rooms (pharmacies) of health facilities, and 6 private pharmacy shops. In total 61 questionnaires were filled.

### Recording of storage temperatures

Temperature data loggers (Tempmate S1 or M1 by imec Messtechnik GmbH, Heilbronn, Germany) were placed at all sites where misoprostol and oxytocin were routinely stored in the visited facilities, i.e. on storage shelves and/or within the refrigerators, both at the maternity ward and in the storage room (pharmacy) if applicable (see Additional File 1). They were fixed in close proximity to the medicines with adhesive tape. The loggers recorded the temperature automatically every 10 min from the time of placement (August or September) to the time of recollection by the study personnel (December; median recording time 130 days). Recorded data was downloaded from the loggers at College of Medicine in Malawi and analysed at Tuebingen University in Germany. The mean kinetic temperature (MKT) was calculated by the imec Messtechnik software.

### Transport and storage of samples

Each sample was labelled with a unique code number using pre-printed adhesive labels, and placed in zip-locked plastic bags at the time of collection. All samples were transported from the collection site to the Pharmacy Department, College of Medicine, Blantyre, in the vehicles of the investigators within the same day; samples labelled for storage at 2–8 °C were transported in a 12 V plug-in refrigerator. They were subsequently stored in the Pharmacy Department in an air-conditioned room (< 25 °C) or in a refrigerator according to the storage requirement stated on the label. Samples were hand-carried to Germany via airplane in an insulating bag (< 24 h transport time) and stored at the Pharmaceutical Institute of Tübingen University in a refrigerator or in an air-conditioned room (< 25 °C) until analysis. In order to document the transport and storage conditions of the samples, temperature loggers were placed with the samples from the day of collection until the day of analysis.

### Sample analysis

All samples were first inspected visually by N.H. at Tübingen University. Oxytocin injections were analysed according to the United States Pharmacopoeia (USP 40, Oxytocin injections) for identity, assay and pH value. The assay was carried out by High Performance Liquid Chromatography (HPLC, Agilent Infinity 1260 II with binary pump, variable wavelength detector, refrigerated autosampler and integrated column compartment; Agilent Technologies, Santa Clara, CA, USA) with mobile phase A (0.1 M NaH_2_PO_4_ buffer) and mobile phase B (acetonitrile: H_2_0 1:1 V/V) using the following gradient: 0 min, 30% B; 10 min, 40% B; 17.5 min, 65% B; 20.5 min, 65% B; 23.5 min, 30% B; 26 min, 30% B. Flow rate was 1.5 ml/min, the injection volume was 70 μl, the column with guard was Reprospher 100 (12.5 cm × 4.6 mm, 5 μm C18; Dr. Maisch GmbH, Tübingen, Germany), and detection was set at 220 nm. From each sample, three vials were analysed independently. USP Reference Standard (batch N° F3K133) was obtained from Merck KGaA (Darmstadt, Germany). Solvents were HPLC grade.

Misoprostol tablets were tested according to the International Pharmacopeia 2017 (Misoprostol tablets) for identity, assay and dissolution, using the HPLC system mentioned above and a mixture of acetonitrile and water (45:55 V/V) as isocratic mobile phase, with a flow rate of 1.5 ml/min, a ReproSil-XR 120 colunm (C18, 5 μm, 150 mm × 4.6 mm; Dr. Maisch GmbH, Tübingen, Germany), injection volume 100 μl (assay) or 250 μl (dissolution), and UV detection at 200 nm. For assay, 5 tablets each were dissolved in 50 ml mobile phase in two independent experiments, and each of the two solutions was analysed twice by HPLC, resulting in four measurements for each sample. If less than 10 tablets were available for the respective sample, the assay was conducted by dissolving 3–5 individual tablets independently in 10 ml mobile phase each and analysing by HPLC, in order to have tablets left for retesting. Misoprostol Ph. Eur. reference standard (batch N° 3.0) was obtained from EDQM (European Directorate for the Quality of Medicines) Strasbourg. If the chromatogram of the assay showed additional peaks, the sample was also tested for related substances according to Kahsay et al. [28].

Dissolution was tested using a dissolution tester PT-WS 610 (Pharma Test Apparatebau AG, Hainburg, Germany). For each sample, six tablets were investigated independently as described in the misoprostol tablets monograph of the International Pharmacopeia, using 500 ml of water R as dissolution medium. A paddle apparatus with 50 rpm was used, and samples were drawn after 30 min through an in-line filter. Dissolution tests were only performed if at least 30 tablets were available for the respective sample.

For both oxytocin and misoprostol assay, and for misoprostol dissolution, 5-point calibration curves were prepared to assure linearity. Assay methods were validated according to USP 40 for system suitability, linearity and precision. Sample analysis was conducted at the Pharmaceutical Institute of Tübingen University, Germany, unblinded to packaging. All samples were within their shelf life at time of analysis, with the exception of one oxytocin sample and two misoprostol samples. Classification as within specification or out of specification was based for oxytocin injections on the specifications of USP 40 for assay and pH value, and for misoprostol tablets based on the specifications of International Pharmacopeia 2017 for assay and dissolution. Assay results between 80 and 90% and 110–120% of the declared content were considered as moderate deviations, contents of less than 80% or more than 120% were considered as extreme deviations [29]. As per definition of WHO, products that deliberately/fraudulently misrepresent their identity, composition or source were considered falsified [30].

### Registration status of medicine brands

PMPB was contacted to enquire the registration status of the medicines collected. If they were registered, the PMPB registration number was requested.

### Statistical analysis

Statistical evaluation was done using JMP 14.2 (SAS GmbH, Heidelberg, Germany). Confidence intervals were calculated using descriptive distribution analysis, significance of differences between storage sites were calculated using one-way analysis of variance (ANOVA), and correlation between age of samples and content was calculated using bivariate analysis. Means and relative standard deviations were calculated using Microsoft Excel 2016 (Microsoft Corporation, Redmond, Washington, USA).

### Information of national authorities and stakeholders

Following the study protocol and WHO guidelines [26], the national medicine regulatory authority of Malawi (PMPB) and the WHO Rapid Alert System were informed immediately about confirmed out-of-specification results representing a serious health risk. After completion of the study and data analysis, the survey results were presented to PMPB, CMST, the Ministry of Health and national and international stakeholders during a meeting in Lilongwe, Malawi, on Sept. 4th, 2019. Additionally, the findings were presented to health workers in the four study districts Ntcheu, Blantyre, Chikwawa and Neno on Sept. 5th, 10th, 11th and 12th, 2019, respectively, including appropriate trainings in cooperation with the Malawi National Pharmacovigilance Centre.

## Results

### Availability of oxytocics

**Fig. 3:**
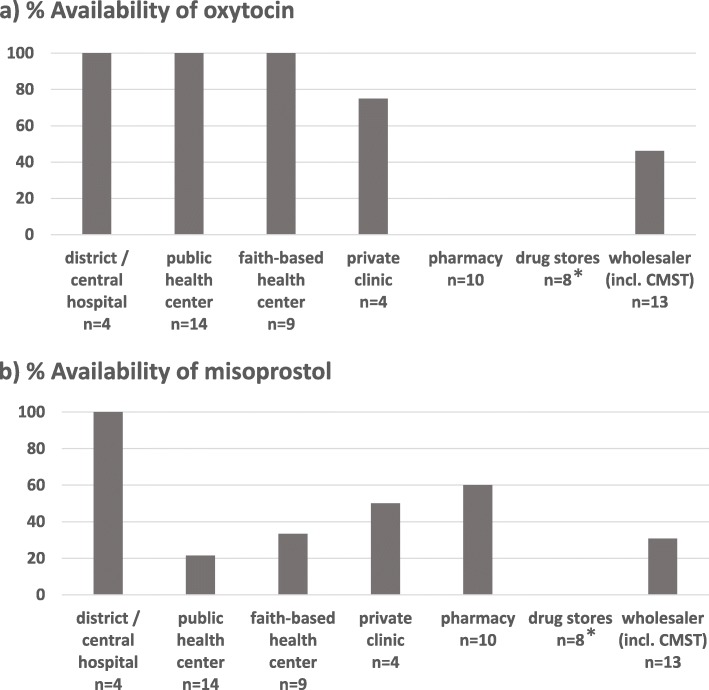
Availability of oxytocics at visited facilities. n = number of facilities. CMST = Central Medical Stores Trust. * Drug stores are not allowed to stock oxytocin and misoprostol, therefore 0% availability was expected in these facilities

Figure 3 shows the availability of oxytocin and misoprostol in the different health facilities and drug outlets included into this study. According to the Malawi Essential Medicines List (MEML) 2015, oxytocin should be available in health centers, district hospital and central hospitals [7]. Indeed, each of the 27 visited hospitals and health centers (both public and faith-based) had oxytocin available. Also, three out of four private clinics had oxytocin in stock; the one not stocking oxytocin did not offer routine delivery services, but did stock misoprostol for abortions. In contrast, none of the private licensed pharmacies had oxytocin in stock.

According to MEML 2015, misoprostol should be available at district and central hospital level [7], but not at health centers unless these have specialised clinical expertise. Indeed, misoprostol was available at all four investigated hospitals, but only in few of the health centers and private clinics. Misoprostol was also available at 6 of the 10 investigated private pharmacy shops.

Two hospitals, one faith-based health center and one private clinic reported to have ergometrine in stock, but none of the public health centers and none of the private pharmacies. Ergometrine maleate is still listed as an oxytocic in the MEML 2015, but is increasingly considered as obsolete.

In the interviews, it was enquired whether stock-outs of oxytocin and misoprostol had been experienced in the last 6 months. In case of public and faith-based health facilities and private clinics, this information was verified by inspecting the stock cards for the respective medicines. 73% of these facilities had not experienced oxytocin stock-outs in the last 6 months, and the rest reported less than 1 month of stock-out time. Out of the 12 health facilities and 6 private pharmacies which had misoprostol in stock at the time of the visit, 11 (61%) reported no stock-outs of misoprostol in the last 6 months, but 3 (17%) reported stock-out times of more than 1 month. Detailed results on the reported stock-out times are shown in Additional File 3.

In public and faith-based health facilities as well as in private clinics, also the amount of oxytocin in stock at the time of the visit was checked, and the consumption over the last 6 months was calculated from the stock cards; this information was available in 29 of the 31 included health facilities. The amount of oxytocin in stock was usually sufficient for 1.4 months (= median value; range: 0.1–25 months; mean: 4.0 months).

In Malawi, drug stores are licensed private outlets which do not require a pharmacist but a pharmacy technician, nurse, or clinical officer on their premises [31]. They are allowed to dispense a limited number of common medicines, but not prescription or pharmacist-only medicines, and therefore not oxytocin or misoprostol. Nevertheless, we decided to include also drug stores, because according to local contact persons some drug stores may illegally stock misoprostol for use in abortions. However, all of the eight drug stores visited by the mystery shoppers in this study stated that they do not sell oxytocin or misoprostol.

### Storage conditions for oxytocics

According to the manufacturers’ information stated on the labels, out of the six misoprostol brands collected in the course of this study, two brands (representing four samples) needed to be stored below 25 °C, three brands (representing 24 samples) below 30 °C, and one brand (representing two samples) showed no information about the storage temperature on the label.

Out of the nine brands of oxytocin collected, five brands (representing 23 samples) were labeled for storage at 2–8 °C, two brands (representing 39 samples) were labeled for storage below 25 °C, and two brands (representing three samples) were labeled for storage below 30 °C (see Tables 1 and 2 and Additional File 1). Therefore, for correct storage of 35% of the oxytocin samples a refrigerator was required. Out of the 31 health facilities (hospitals, health centers and private clinics), one public health center and two faith-based health centers stated to have no functioning refrigerator. While two of these three facilities correctly used oxytocin brands which, according to the label, did not require refrigerated storage, one was found to stock a brand which required storage in a refrigerator (Additional File 1, facility no. 17).

**Table 1:**
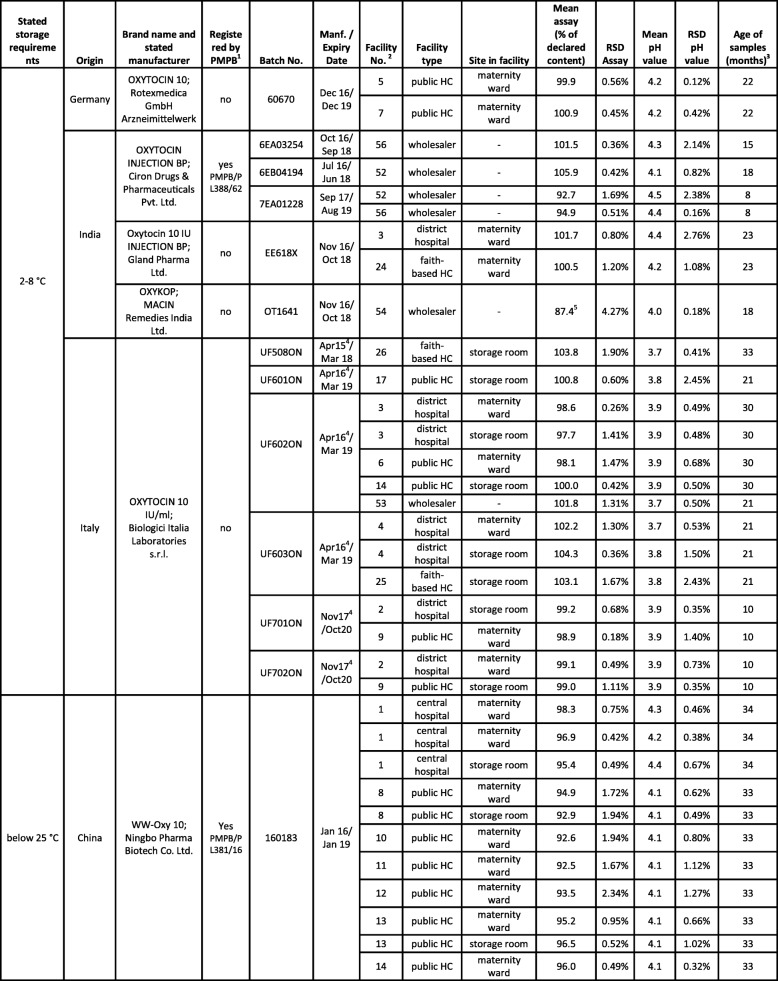

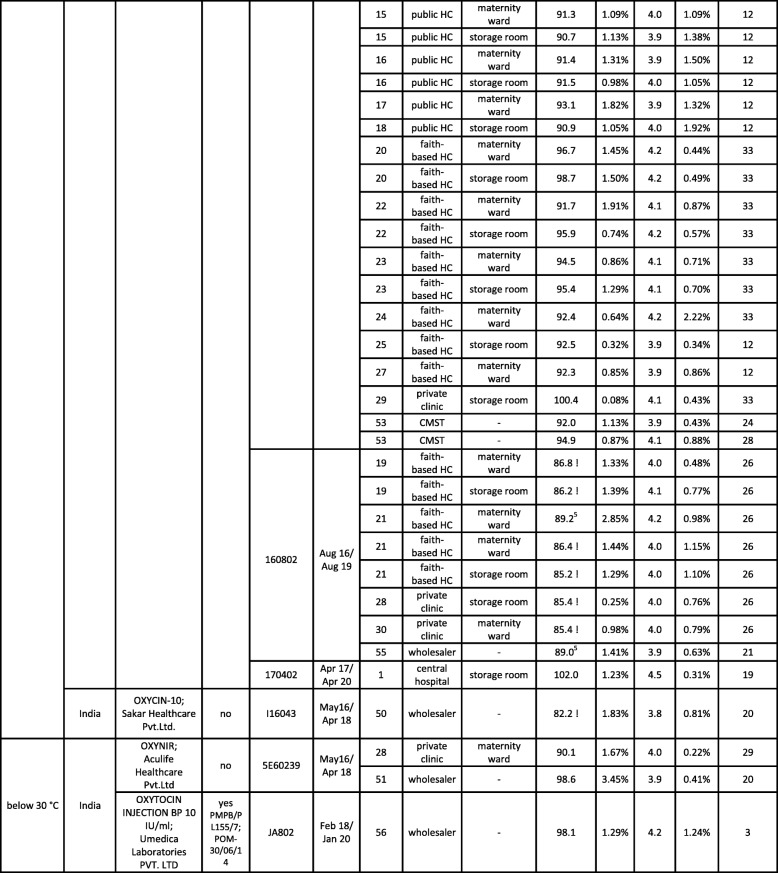
List of all oxytocin samples collected in the course of this study. Assay values which are out of specification are marked by “!”. RSD = relative standard deviation; HC = Health Center; CMST = Central Medical Stores Trust

^1^*PMPB = Pharmacy, Medicine and Poisons Board of Malawi; for registered medicines, the PMPB registration number is given;*^*2*^*facility number as listed in* Additional File 1*;*^*3*^*at time of analysis;*^*4*^*manufacturing date not stated on packaging; information from the websites of MHRA (**http://www.mhra.gov.uk**) and HPRA (**http://www.hpra.ie/**);*^*5*^*assay value out of specification, but deviation from the 90% threshold not statistically significant considering the standard deviation of the measurement*

**Table 2:**
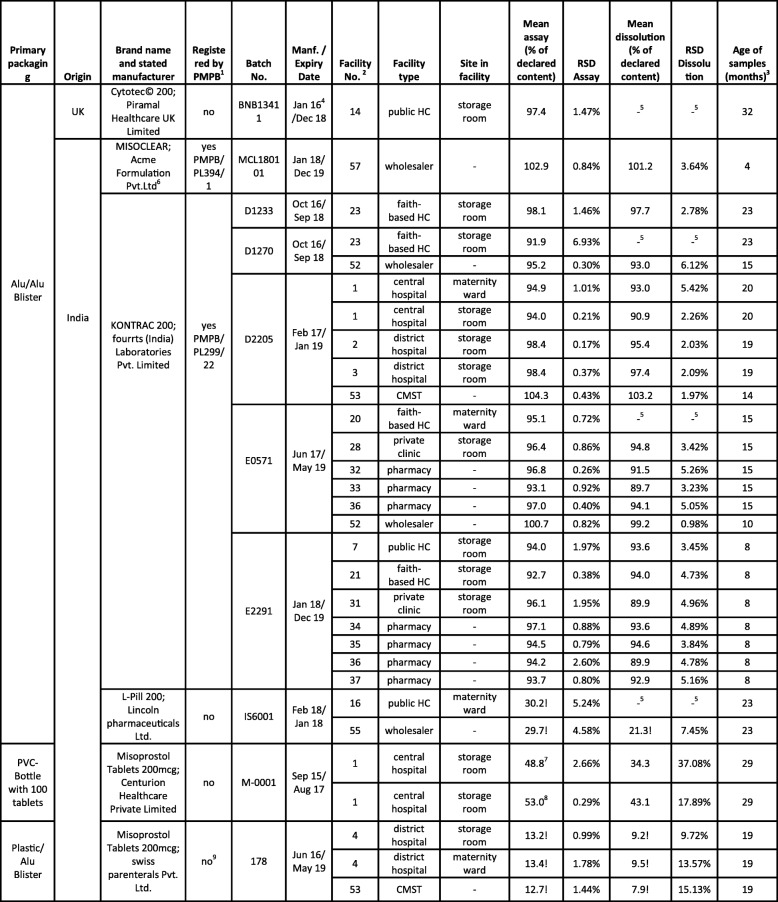
List of misoprostol samples. Assay and dissolution values which are out of specification are marked by “!”. RSD = relative standard deviation; HC = Health Center; CMST = Central Medical Stores Trust

^1^*PMPB = Pharmacy, Medicine and Poisons Board of Malawi; for registered medicines, the PMPB registration number is given;*^*2*^*facility number as listed in* Additional File 1*;*^*3*^*at time of analysis;*^*4*^*manufacturing date not stated on packaging; information from the websites of HPRA (**www.hpra.ie**) and emc (**www.medicines.org.uk/emc**);*^*5*^*no dissolution testing due to small sample size;*^*6*^*WHO-prequalified finished pharmaceutical product* [32, 33]*;*^*7*^*collected in Sep 17 as open PVC-bottle, analysed in Feb 18;*^*8*^*collected in Sep 17 as sealed PVC-bottle, analysed in Feb 18;*^*9*^*incomplete information; manufacturer may have won a tender with CMST and may therefore have undergone accelerated registration*

All of the six licenced pharmacies where the interview was conducted stated to have a functioning refrigerator.

All refrigerators were operated with electricity. In 41% of the visited facilities the health worker stated that there were power failures every day. Only 51% of the facilities reported to have generators or solar panels as potential back-ups in case of power failures, while 27% stated that there were no measures taken to maintain appropriate storage conditions in the event of power failures.

Out of the 17 oxytocin samples labelled for storage at 2–8 °C and collected at health facilities, 13 had been correctly stored in a refrigerator, while four had been stored at ambient temperature, in violation of correct storage procedures. These four samples had been collected in public health centers in Blantyre, Chikwawa and Ntcheu. At one of these sites, the responsible person answered to the question how oxytocin should be stored “at room temperature”, indicating a lack of knowledge of correct storage procedures. Notably, at most facilities there was only one stock card for oxytocin, regardless of the presence of different oxytocin brands with different storage requirements and therefore different storage locations.

Out of the 36 oxytocin samples labelled for storage at < 25 °C or < 30 °C and collected at health facilities, 29 had indeed been stored at ambient temperature. The other 7 had been stored in the refrigerator, which is no problem for oxytocin stability and therefor no violation of correct storage procedures.

To determine actual storage temperature, temperature data loggers were placed at all sites where misoprostol and oxytocin was stored in the facilities (both at the maternity ward and in the storage room if applicable), and temperature was recorded for approximately 4 months (see Methods). Additional File 1 shows the mean kinetic temperature (MKT) recorded at each site. MKT, rather than the simple arithmetic mean of the recorded temperatures, is the relevant measurement for the stability of pharmaceuticals [34].

The MKT recorded at non-refrigerated storage sites ranged from 21.4 to 31.0 °C (median: 26.2 °C). As expected, Chikwawa was the hottest district with a median MKT of 28.1 °C. The highest single temperature measurement (40.1 °C) was also recorded in a facility in Chikwawa.

35 of the oxytocin samples collected in health facilities (hospitals, health centers and private clinics) were labelled for storage below 25 °C. However, this temperature was exceeded in 16 of the places where these samples had been collected from, and the median MKT in these 16 places was 27.3 °C.

Only three oxytocin samples collected in the course of this study were labelled for storage below 30 °C (one collected in a private clinic, two from wholesalers). However, even this temperature was exceeded in 4 of the facilities visited in this study (median MKT in these four places = 30.2 °C; see Additional File 1).

17 of the oxytocin samples collected in health facilities were labelled for storage at 2–8 °C. As mentioned above, four of these samples were incorrectly stored outside the refrigerator. But also for five of the respective storage sites inside refrigerators, MKTs between 10.6–18.3 °C were recorded (see Additional File 1). Three of these were recorded at maternity wards who reported that there were power failures every day.

### Interviews with health workers

Using a questionnaire, interviews were conducted with the persons responsible for storage and /or administration of oxytocics in the respective facilities (see Methods). In these interviews, only 24% of the persons responsible for the storage room (pharmacy), and 32% of the persons responsible for administration of oxytocics in the maternity wards, reported to have received a training on storage, distribution and handling of cold chain medications. Out of 61 interviewed persons, 7 reported that they had observed ineffective oxytocin (*n* = 5) or ineffective misoprostol (*n* = 2) in their professional practice. Notably, only 2 of these 7 stated that they had notified the authorities (or the suppliers) about this.

Standard Treatment Guidelines (STGs) for oxytocin or misoprostol were available at 23 (74%) out of the 31 visited health facilities, and at 66% of the visited maternity wards. At maternity wards, all interviewed health workers correctly stated that oxytocin for prevention or treatment of PPH is given “always after delivery of the child”. Further results from the interviews are summarized in Additional File 3.

### Overview of collected oxytocin samples

**Fig. 4:**
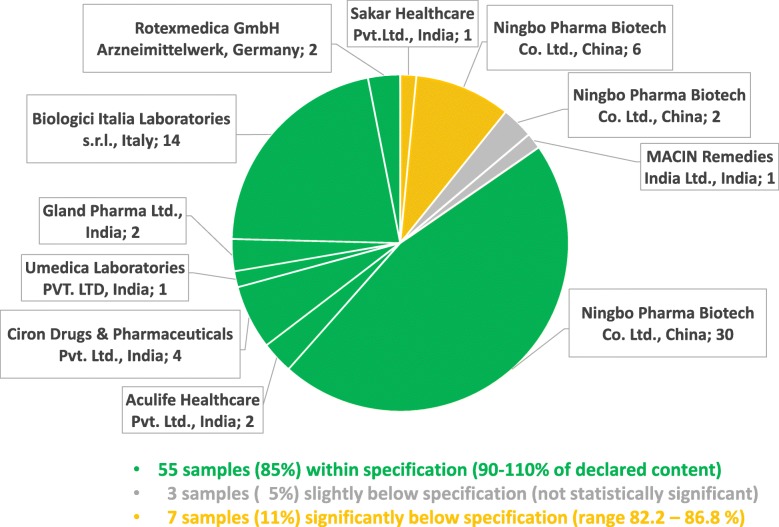
Assay results of oxytocin samples from different manufacturers

Table 1 lists the 65 oxytocin samples collected in the course of this study. As depicted in Fig. 4, the two most frequently encountered preparations were a branded generic preparation from China labelled for storage at < 25 °C (38 samples, three batches), and a generic preparation from Italy labelled for storage at 2–8 °C (14 samples, six batches). Both were distributed by the government-operated Central Medical Stores Trust (CMST) to the public (and partly also faith-based) health facilities. The remaining 13 samples represented 7 different brands (nine batches) from India and from Germany. Most of these were collected outside of the government health facilities.

The total shelf life of the samples declared by the manufacturer varied from 2 to 4 years. Only a single oxytocin sample was expired at time of collection; it was found in the maternity ward of a private clinic.

Out of the 9 brands collected, only 3 were registered by the Pharmacy, Medicines and Poisons Board (PMPB) of Malawi, 6 were not (Table 1).

### Chemical analysis of oxytocin samples

Visual inspection showed a surprisingly high number of spelling errors in the leaflet of the oxytocin brand produced in China, but otherwise gave no indication of quality defects or falsification. The content of the oxytocin samples was determined by HPLC and the pH value was measured, following the procedures of USP 40 (see Methods). According to USP 40, oxytocin injections have to contain between 90 and 110% of declared amount of oxytocin, and to show a pH value between 3.0 and 5.0.

Table 1 shows the pH and assay values determined for all samples. The pH value was found to be within specification for all samples. 55 of the 65 samples (85%) also complied with the specification for the oxytocin content (Fig. 4). Three samples showed assay values slightly below 90%, but their deviations from the 90% threshold were not statistically significant considering the standard deviation of the measurement. Seven samples (11%) showed oxytocin contents which were significantly lower than 90% of the declared content (range 82.2–86.8%). One of the seven failed samples was from an Indian manufacturer, while the other six derived from a single batch produced by the Chinese manufacturer whose preparations had already been noted for spelling errors in the leaflet. Two other batches from that same Chinese manufacturer were found to be within specification, including a batch with a shorter remaining shelf-life than the failed samples.

**Fig. 5:**
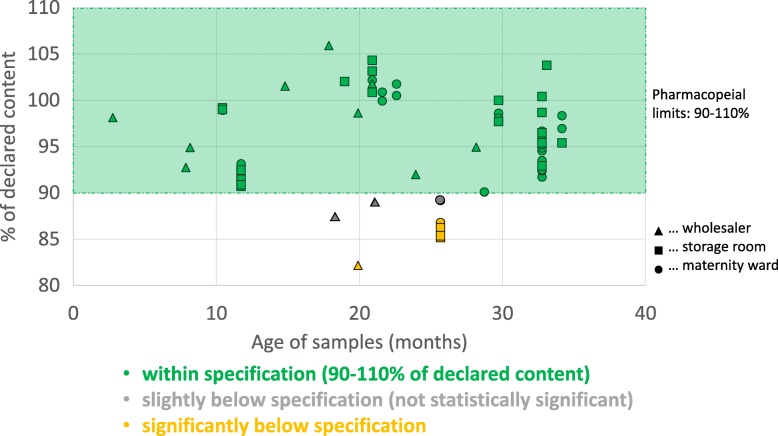
Oxytocin content in relation to age and collection site of the samples (*n* = 65). The age of the sample denotes the time between manufacturing date and date of analysis in months

The age of the samples at time of analysis (i.e. the time elapsed after their manufacturing date) varied from three to 34 months. As shown in Fig. 5, there was no correlation between oxytocin content and age of samples (r^2^ = 0.00024; *p* = 0.90). The mean content of samples collected from wholesalers was 94.9%, while the mean content of samples from the storage rooms (pharmacies) of health facilities was 96.0%, and from maternity wards 94.7%. Therefore, no relevant differences were observed between these groups.

As mentioned above, four samples of oxytocin labelled for storage at 2–8 °C had been found to be incorrectly stored at ambient temperatures. Nevertheless, all of these were within specification (98.1–100.8% of declared content). Seven samples labelled for storage below 25 or below 30 °C had been found to be stored in the refrigerator. Nevertheless, two of these seven samples were out of specifications.

None of the 23 samples labelled by the manufacturer for storage at 2–8 °C was found to be out of specification. In notable contrast, seven of the 39 samples labelled for storage < 25 °C failed quality testing, showing insufficient oxytocin content. Only three samples labelled for storage < 30 °C were found in this study, and none of these failed quality testing.

Notably, all 32 oxytocin samples collected from government health facilities were within specifications. Of the seven failed samples, four had been collected in faith-based health centers, two in private clinics, and one from a wholesaler.

### Overview of collected misoprostol samples

**Fig. 6:**
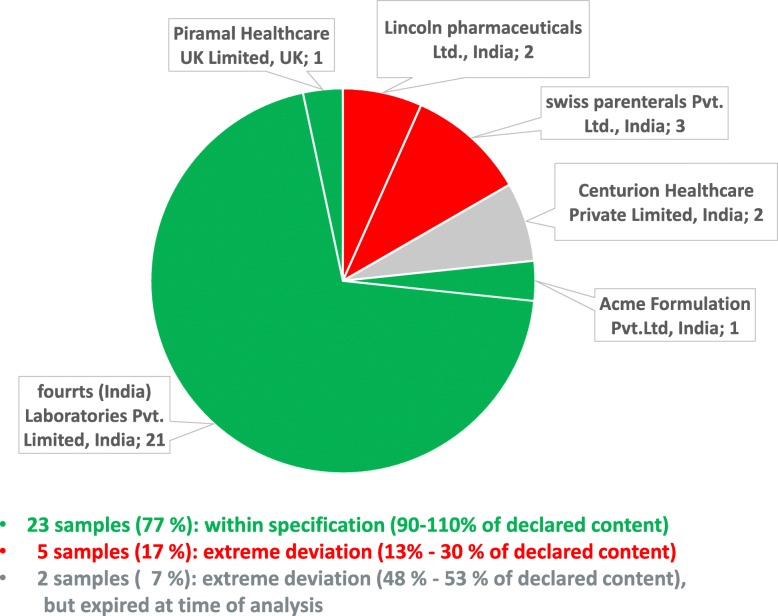
Assay results of misoprostol samples from different manufacturers

Table 2 lists the 30 misoprostol samples collected in the course of this study. As depicted in Fig. 6, all but one of these samples were manufactured in India. The most frequently encountered brand was a branded generic preparation (21 samples, 5 batches) correctly packaged in aluminium-aluminium blisters and distributed by CMST, as well as by a private wholesaler. Three further brands were also correctly packaged in aluminium-aluminium blisters: one originator brand from the UK (one sample) and two branded generics from India (3 samples, 2 batches); one of these latter ones represented a WHO-prequalified product (see Table 2). Another branded generic from India (3 samples, 1 batch) was incorrectly packaged in aluminium-plastic blisters; it was collected both at CMST and in a district hospital. One further generic preparation from India (2 samples, 1 batch) was even packaged in screw-cap PVC bottles containing 100 tablets, offering no protection of the individual tablets against humidity once the bottle is opened. Such packaging is grossly inadequate for misoprostol tablets. This brand was found in the pharmacy of the central hospital. The two samples of this brand (one sealed bottle and one already opened bottle) had just expired in the month before collection, and had correctly been quarantined by the facility. In view of the highly unusual packaging, it was decided to collect and analyse them nevertheless.

None of the other 28 samples was expired. The total shelf life declared by the manufacturers varied from two to 3 years.

Out of the six brands collected, only two were registered by PMPB in Malawi, four were not (Table 2).

### Chemical analysis of misoprostol samples

Visual inspection showed the above-mentioned shortcomings of the packaging of several samples, but otherwise gave no indication of quality defects or falsification. The content of the misoprostol samples, as well as dissolution of the active pharmaceutical ingredient (API), was determined by HPLC following the procedures of the International Pharmacopeia 2017 (see Methods). According to the International Pharmacopeia, misoprostol tablets have to contain between 90 and 110% of the declared content, and at least 80% of the API must dissolve within 30 min under the conditions described in the pharmacopeia.

Table 2 shows the assay and dissolution values determined for all samples. 23 of the 30 samples (77%) complied with the specification for the misoprostol content (Fig. 6), and all of these also complied with the specification for dissolution. Notably, all compliant samples were packaged in aluminium-aluminium blisters.

In contrast, both samples of the brand packaged in PVC bottles and all three samples of the brand packaged in plastic-aluminium blisters were found to be extremely out of specification, but also one brand packaged correctly in aluminium-aluminium blisters (Table 2).

The brand packaged in PVC bottles contained only 53.0% (sealed bottle) and 48.8% (opened bottle) of the declared misoprostol content. Especially the tablets in the opened bottle also showed poor dissolution (34.3% of the declared content), with an extremely high standard deviation between the individual tested tablets (Table 2). This suggests that the quality of the tablets had been severely affected by (unequal) exposure to humidity. Since these tablets were expired at the time of analysis, these results do not represent definitive proof of poor quality before expiry, though it appears very likely that they had been already out of specification before reaching their expiry date.

As mentioned, one brand (by Lincoln Pharmaceuticals Ltd., India) which was packaged in aluminium-aluminium blisters was nevertheless extremely out of specification (Table 2), containing only 29.7 or 30.2% of the declared misoprostol amount.

The worst brand discovered in the course of this study was manufactured in India by Swiss Parenterals PVT, Ltd. It was packaged in plastic-aluminium blisters. HPLC analysis revealed a misoprostol content of only 12.7–13.4% of the declared amount, and only 7.9–9.5% of the declared content was found to dissolve. Testing of the extremely substandard misoprostol preparations for related substances (Additional File 4) showed the typical degradation products named in Fig. 1. Obviously, it cannot be decided whether all of the degradation occurred after manufacturing of the tablets, or whether the tablets were manufactured using an already partially degraded API.

The age of the misoprostol samples at time of analysis was between four and 32 months.

### Product recall in Malawi, and closure of the responsible distributor in the United Kingdom

The extremely substandard misoprostol preparations by Lincoln Pharmaceuticals Ltd. and Swiss Parenterals PVT Ltd. were discovered in the first part of this study, by sample collection in September 2017. As foreseen in the study protocol, PMPB, CMST and WHO were informed immediately about this finding. PMPB thereupon issued a product recall, and CMST discontinued supplying the substandard misoprostol. In the subsequent main part of this study, with sample collection in August 2018, no further substandard misoprostol samples were discovered, indicating that the recall had been effective.

The misoprostol preparation by Swiss Parenterals PVT Ltd. was labelled as “Distributed by Premiumway International. U.K. www.premiumway.co.uk”. Our finding of the poor quality of this preparation was also reported in the electronic newsletter of the initiative “e-drug” [35]. In August 2018 it was pointed out by the moderator of that newsletter that, according to the UK government website Companies House (https://beta.companieshouse.gov.uk/), the distributor Premiumway International was located at the same address, and managed by the same directors, as the company Unimed International Ltd. who had been reported to distribute poor-quality propofol (an anaesthetic medicine) to the government of Zambia [36, 37]. In the ensuing correspondence, WHO asked the authors of this paper to send a sample of the misoprostol tablets distributed by Premiumway International to the British medicine regulatory authority (MHRA) for confirmatory analysis. The precise actions taken subsequently by MHRA have not been released to the public, but as published on the UK government website Companies House, both Premiumway International and Unimed International Ltd. went into voluntary liquidation on 28. January 2019.

## Discussion

The present study showed good availability of oxytocics, especially of oxytocin, in the health facilities of Malawi. On the other hand, it revealed widespread problems with the maintenance of correct medicine storage temperatures. Nevertheless, the majority of the investigated oxytocics showed good quality. However, 11% of the oxytocin samples showed moderate deviations from specification (containing 82.2–86.8% of the declared amount of the API), and 17% of the misoprostol samples even showed extreme deviations (containing only 12.7–30.2% of the declared amount of API). The latter finding represents a serious risk to patient safety in maternal health care. Notably, both national and international authorities reacted swiftly and correctly to this finding.

The excellent oxytocin availability in the investigated government and faith-based health facilities is an important contribution to the attainment of the SDGs, especially to the reduction of global maternal mortality. Our finding is consistent with data from the Malawi Service Provision Assessment 2013–14, according to which 95% of the facilities (hospitals, health centers, clinics, dispensaries, health posts) which offered delivery services had oxytocin available [38].

Nearly half of the oxytocin and misoprostol samples collected in this study were labelled for storage below 25 °C. Using temperature loggers which automatically recorded the storage temperatures over approximately 4 months, our study clearly proved that this storage requirement cannot be complied with in many health facilities in Malawi. Improvements in the construction of storage rooms may reduce this problem, but air conditioning is most likely not an economically and practically feasible solution in rural health facilities of a low-income country such as Malawi. One feasible solution may be the procurement of medicines which have been proven to be stable at storage temperatures of up to 30 °C, as recommended by WHO for very hot countries (climatic zones III and IVA/IVB) but currently not for Malawi [39]. We recorded mean kinetic temperatures even higher than 30 °C in four health facilities in Malawi (Additional File 1). However, the 30 °C threshold was only exceeded by small margins (measured MKTs: 30.1, 30.2, 30.2 and 31.0 °C), and the period of our measurements (August/September until December) included the hottest season in Malawi.

Many currently available oxytocin preparations are labelled for storage at 2–8 °C. Just as previous studies in other countries [16, 40], our investigation showed that this storage temperature could not be reliably maintained in several health facilities in Malawi. Reasons for this included lack of refrigerators, frequent power failures, and lack of back-up generators or solar panels in many facilities. In this situation, it appears tempting to replace the oxytocin brands requiring storage at 2–8 °C by preparations labelled for storage at higher temperatures, or even by misoprostol tablets which do not require refrigeration. Unfortunately, our study suggests that this strategy does not offer a straightforward and reliable solution of the problem. Notably, all collected oxytocin samples labelled for storage at 2–8 °C were found to be within specifications, even when incorrectly stored in the health facility. In sharp contrast, 18% of the oxytocin preparations labelled for storage below 25 °C were found to be out of specification. A heat stable formulation of carbetocin, an oxytocin analogue, has just recently been added to the 2019 Model List of Essential Medicines of the World Health Organisation (WHO) and may become an alternative to other oxytocics [41]. However, it is not yet included into the Malawi Essential Medicines List, and we did not encounter any carbetocin samples in the course of our study.

In this study, only a small number of different oxytocin brands was found and investigated, therefore a generalization of the present results to the global situation may not be possible. But clearly, the present study shows an urgent need to reconfirm the stability claims of oxytocin preparations labelled for room temperature storage. Furthermore, it shows the importance of the procurement of good quality medicines from reliable manufacturers. This applies not only to oxytocin but even more to misoprostol, of which at least two extremely substandard brands were found in circulation in Malawi.

One option to ensure good quality is to restrict procurement to WHO-prequalified medicines [32, 33] and medicines produced in countries with a stringent regulatory authority (SRA) [42]. Of the 95 oxytocin and misoprostol samples collected in the present study, one (Misoclear®) represented a WHO-prequalified product and 17 were manufactured in countries with an SRA. Notably, none of these 18 samples was out of specification. On the other hand, also 5 brands of oxytocin and misoprostol manufactured in countries without an SRA, and not prequalified by WHO, comprised no samples which were out of specification. This demonstrates the importance of supplier prequalification in medicine procurement. The Medical Abortion Commodities Database (www.medab.org) has listed misoprostol products likely to be of good quality, as well as information on their availability. Of the six misoprostol preparations investigated in this study, two are listed in this database, and indeed these were found to be of good quality (i.e. within compendial specifications).

As mentioned above, the total number of different oxytocic preparations encountered in this study was small (9 oxytocin brands, 6 misoprostol brands). This may be a reflection of the small market size of Malawi and the resulting reluctance of manufacturers and international distributors to engage in medicine sales in this country. Unfortunately, this reluctance may severely restrict the possibilities to procure affordable, good-quality medicines. In spite of the above-mentioned problems, 11% of oxytocin samples investigated in this study were out of specification, much less than the 57.5% out-of-specification rate of oxytocin samples collected in Africa reported in a review of studies from 15 LMICs [20], and also than the 74.2% reported from a study in Nigeria [16]. In the latter study, all oxytocin samples were tested for identity, assay, pH value, sterility, and fill volume; all failing samples failed due to the assay values, while no samples failed in any of the other criteria [16].

All oxytocin samples collected in government health facilities in Malawi were of good quality, and the Malawi public health services deserve praise for this achievement, despite the observed problems with the quality of some misoprostol preparations.

The seven failing oxytocin samples showed API contents between 82.2 and 86.8% of the declared content. Following the terminology of an authoritative WHO study on medicine quality [29], we classified these as “moderate deviations” and marked them in Fig. 4 in yellow colour. Even moderate deviations from medicine specifications are not acceptable and need to be ruled out by appropriate measures. Nevertheless, the risk to patient safety posed by these oxytocin preparations is probably limited. However, this situation is clearly different for the five extremely substandard misoprostol preparations identified in this study, which are marked in red in Fig. 6. They contained only 12.7–30.2% of the declared content of misoprostol, and treatment failures will result from such extremely substandard medicines.

Misoprostol is an exceptionally instable API, and misoprostol tablets must therefore be produced competently using appropriate stabilizing agents. To improve misoprostol stability, a 1% dispersion of the API in hydroxypropylmethylcellulose (HPMC) is usually used [18, 43, 44]. Furthermore, a recent publication in WHO Drug Information [11] clearly demonstrated the supreme importance of aluminium-aluminium blisters for packaging of misoprostol tablets and for their protection from humidity. However, our finding of a misoprostol brand using plastic-aluminium blisters, and even of a brand using a PVC bottle with no individual blistering of the tablets, shows that this information is not yet sufficiently applied in misoprostol manufacturing and procurement practice.

## Conclusions

The availability of oxytocin in the four investigated districts of Malawi was found to be very good, and its quality was notably better than reported in previous studies carried out in other LMICs. However, storage conditions for oxytocin and misoprostol at the health facilities often did not meet the requirements stated by the manufacturers on the labels. The observed occurrence of substandard oxytocics apparently resulted both from shortcomings in the manufacturing process, including inappropriate formulation and packaging, and from deterioration during storage, accelerated by inappropriate storage conditions. Yet, this small study could not supply evidence at which part of the supply chain deterioration primarily occurs. Extremely substandard misoprostol tablets were found which represented a serious risk to maternal health. This shows the need for continued efforts for quality assurance in medicine procurement and registration, as well as for post-marketing surveillance within a functioning pharmacovigilance system. The case of the extremely substandard misoprostol tablets also highlights the importance of the immediate reporting of substandard medicines to national authorities, international stakeholder and the medical community, for a further improvement of patient safety within the country and worldwide.

## Supplementary information

**Additional file 1.** List of included health facilities, pharmacies and wholesalers, with sizes of collected misoprostol and oxytocin samples, and storage conditions.

**Additional file 2.** Questionnaire and consent form.

**Additional file 3.** Results from Interviews.

**Additional file 4.** Related substances chromatogram of Misoprostol Tablets 200mcg by swiss parenterals Pvt. Ltd.

## Data Availability

The datasets used and/or analysed during the current study are included in this published article and its supplementary information files.

## Abbreviations

ANOVA: Analysis of variance
API: Active pharmaceutical ingredient
CMST: Central Medical Stores Trust
COMREC: College of Medicine Research and Ethics Committee
GIZ: Gesellschaft für Internationale Zusammenarbeit GmbH
HPLC: High performance liquid chromatography
HPMC: Hydroxypropylmethylcellulose
LMIC: Low- and middle-income country
MEDQUARG: Medicine Quality Assessment Reporting Guidelines
MEML: Malawi Essential Medicines List
MHRA: Medicines and Healthcare products Regulatory Agency, U.K
MKT: Mean kinetic temperature
MSTG: Malawi Standard Treatment Guidelines
PMPB: Pharmacy, Medicines and Poisons Board
PPH: Post-partum haemorrhage
RSD: Relative standard deviation
SDG: Sustainable Development Goal
SOP: Standard operating procedure
SRA: Stringent Regulatory Authority
STG: Standard Treatment Guideline
UN: United Nations
UNCoLSC: UN Commission on Life-Saving Commodities for Women and Children
USAID: United States Agency for International Development
USP: United States Pharmacopoeia
WHO: World Health Organization
