# Supplementary material for: Quality, availability and storage conditions of oxytocin and misoprostol in Malawi
Source: BMC Pregnancy Childbirth. 2020 Mar 29;20:184. doi: 10.1186/s12884-020-2810-9 (PMC7104524; doi:10.1186/s12884-020-2810-9)
Supplement: Supplementary file 1 — Additional file 1. List of included health facilities, pharmacies and wholesalers, with sizes of collected misoprostol and oxytocin samples, and storage conditions. [file 12884_2020_2810_MOESM1_ESM.docx]

| **­­Facility number** | **Type of facility** | **District** | **Sampling site** | **N° of collected misoprostol tablets per sample** | **Misoprostol storage conditions stated on label** | **N° of collected oxytocin vials per sample** | **Oxytocin storage conditions stated on label** | **Oxytocin storage conditions in health facility** | **Mean kinetic room temperature measured over 3 months (°C)** | **Mean kinetic refrigerator temperature measured over 3 months (°C)** | **Questionnaire completed? (yes/no)** |
| --- | --- | --- | --- | --- | --- | --- | --- | --- | --- | --- | --- |
| 1 | central hospital | Blantyre | maternity ward | 40 | < 30 °C | 5  5 | < 25°C  < 25°C | RT  RT | 24.9 | n.rf.s. | yes |
|  |  |  | storage room | 96 100 50 | not stated  not stated  < 30 °C | 5  5 | < 25°C  < 25°C | RT  RT | 24.5^a^; 22.6^b^ | n.rf.s. | yes |
| 2 | district hospital | Chikwawa | maternity ward | - |  | 10 | 2-8°C | refrigerator | n.rt.s. | 14.4 ! | yes |
|  |  |  | storage room | 30 | < 30°C | 10 | 2-8°C | refrigerator | 28.1 | 13.2^c^ | yes |
| 3 |  | Neno | maternity ward | - |  | 10  10 | 2-8°C  2-8°C | refrigerator  refrigerator | n.rt.s. | 11.9 ! | yes |
|  |  |  | storage room | 50 | < 30°C | 10 | 2-8°C | refrigerator | 21.9 | 2.8 | yes |
| 4 |  | Ntcheu | maternity ward | 38 | < 25 °C | 10 | 2-8°C | refrigerator | 26.5 ! | 3.6 | yes |
|  |  |  | storage room | 50 | < 25 °C | 10 | 2-8°C | refrigerator | 21.5 | 3.6 | yes |
| 5 | public health center | Blantyre | maternity ward | - |  | 10 | 2-8°C | refrigerator | n.rt.s. | 4.5 | yes |
| 6 |  | Blantyre | maternity ward | - |  | 8 | 2-8°C | RT ! | 26.2 | n.rf.s. | yes |
| 7 |  | Blantyre | maternity ward | - |  | 10 | 2-8°C | refrigerator | 23.3 | not measured | yes |
|  |  |  | storage room | 50 | < 30°C | - | - | - | 23.6 | n.rf.s. | yes |
| 8 |  | Chikwawa | maternity ward | - |  | 10 | < 25°C | refrigerator! | n.rt.s. | 18.3 ! | yes |
|  |  |  | storage room | - |  | 10 | < 25°C | RT | 23.7 | n.rf.s. | yes |
| 9 |  | Chikwawa | maternity ward | - |  | 9 | 2-8°C | RT ! | not measured | n.rf.s. | yes |
|  |  |  | storage room | - |  | 10 | 2-8°C | RT ! | 29.2 | n.rf.s. | yes |
| 10 |  | Chikwawa | maternity ward | - |  | 6 | < 25°C | RT | 28.3 ! | n.rf.s. | yes |
|  |  |  | storage room | - |  | - | - | - | 27.7^a^; 27.9^b^ | n.rf.s. | yes |
| 11 |  | Neno | maternity ward | - |  | 10 | < 25°C | RT | 27.4 ! | n.rf.s. | yes |
| 12 |  | Neno | maternity ward | - |  | 10 | < 25°C | refrigerator! | n.rt.s. | 5.3 | yes |
| 13 |  | Neno | maternity ward | - |  | 10 | < 25°C | RT | 30.2 ! | n.rf.s. | yes |
|  |  |  | storage room | - |  | 10 | < 25°C | RT | 28.2 ! | n.rf.s. | yes |
| 14 |  | Neno | maternity ward | - |  | 10 | < 25°C | refrigerator! | n.rt.s. | missing logger | yes |
|  |  |  | storage room | 10 | < 30 °C | 10 | 2-8°C | refrigerator | 26.2 | 6 | yes |
| 15 |  | Ntcheu | storage room | - |  | 10 | < 25°C | RT | 24.2 | n.rf.s. | yes |
|  |  |  | maternity ward | - |  | 10 | < 25°C | RT | 27.4 ! | n.rf.s. | yes |
| 16 |  | Ntcheu | maternity ward | 10 | < 30 °C | 4 | < 25°C | RT | 28 ! | n.rf.s. | yes |
|  |  |  | storage room | - |  | 4 | < 25°C | RT | not measured | n.rf.s. | yes |
| 17 |  | Ntcheu | maternity ward | - |  | 10 | < 25°C | RT | 22.8 | n.rf.s. | yes |
|  |  |  | storage room | - |  | 10 | 2-8 °C | RT ! | 23 | n.rf.s. | yes |
| 18 |  | Ntcheu | storage room | - |  | 10 | < 25°C | RT | 23.1 | n.rf.s. | yes |
| 19 | faith-based health center | Blantyre | maternity ward | - |  | 10 | < 25°C | RT | 24.7 | n.rf.s. | no |
|  |  |  | storage room | - |  | 10 | < 25°C | RT | 25.4 ! | n.rf.s. | yes |
| 20 |  | Blantyre | maternity ward | 13 | < 30 °C | 10 | < 25°C | RT | 26.8^a^; 26.2^b^ ! | n.rf.s. | yes |
|  |  |  | storage room | - |  | 10 | < 25°C | RT | 26.6 ! | n.rf.s. | yes |
| 21 |  | Chikwawa | maternity ward | - |  | 10  3 | < 25°C  < 25°C | refrigerator!  RT | 26.5 ! | 11.8^c^ | yes |
|  |  |  | storage room | 19 | < 30 °C | 10 | < 25°C | refrigerator! | 30.2 ! | 13.8 ! | yes |
| 22 |  | Neno | maternity ward | - |  | 10 | < 25°C | RT | 25.5 ! | n.rf.s. | yes |
|  |  |  | storage room | - |  | 10 | < 25°C | RT | 26.8 ! | n.rf.s. | yes |
| 23 |  | Neno | maternity ward | - |  | 10 | < 25°C | RT | 28.8 ! | n.rf.s. | yes |
|  |  |  | storage room | 20  30 | < 30 °C  < 30 °C | 10 | < 25°C | RT | 29.1^a^;30.1^b^ ! | n.rf.s. | yes |
| 24 |  | Neno | maternity ward | - |  | 10  10 | 2-8°C  < 25°C | refrigerator  RT | 21.4 | 4.9 | yes |
|  |  |  | storage room | - |  | - | - | - | n.rt.s. | n.rf.s. | yes |
| 25 |  | Ntcheu | storage room | - |  | 10  10 | 2-8°C  < 25°C | refrigerator  RT | 24 | 4 | yes |
|  |  |  | maternity ward | - |  | - | - | - | n.rt.s | n.rf.s. | yes |
| 26 |  | Ntcheu | storage room | - |  | 10 | 2-8°C | refrigerator | n.rt.s. | 4.3 | yes |
|  |  |  | maternity ward | - |  | - | - | - | 23.5 | n.rf.s. | yes |
| 27 |  | Ntcheu | maternity ward | - |  | 10 | < 25°C | RT | 27.2 ! | n.rf.s. | yes |
|  |  |  | storage room | - |  | - | - | - | 28.3 | n.rf.s. | yes |
| 28 | private clinic | Blantyre | maternity ward | - |  | 10 | < 30°C | refrigerator! | n.rt.s. | 10.6 ! | yes |
|  |  |  | storage room | 60 | < 30 °C | 10 | < 25°C | RT | 26.4 ! | n.rf.s. | yes |
| 29 |  | Blantyre | maternity ward | - |  | - | - | - | n.rt.s. | n.rf.s. | yes |
|  |  |  | storage room | - |  | 10 | < 25°C | RT | 12.4^d^ | n.rt.s. | yes |
| 30 |  | Chikwawa | maternity ward | - |  | 9 | < 25°C | refrigerator! | n.rt.s. | 5.5 | yes |
|  |  |  | storage room | - |  | - | - | - | n.rt.s. | n.rf.s. | yes |
| 31 |  | Chikwawa | storage room | 60 | < 30 °C | - | - | - | 31 ! | n.rf.s. | yes |
| 32 | private  pharmacy | Blantyre | storage room | 23 | < 30 °C | - | - | - | 24.6 | n.rf.s. | yes |
| 33 |  | Blantyre | storage room | 50 | < 30 °C | - | - | - | 24 | n.rf.s. | yes |
| 34 |  | Blantyre | storage room | 30 | < 30 °C | - | - | - | 25.4 | n.rf.s. | yes |
| 35 |  | Blantyre | storage room | 30 | < 30 °C | - | - | - | 26.5 | n.rf.s. | yes |
| 36 |  | Blantyre | storage room | 36  20 | < 30 °C  < 30 °C | - | - | - | not measured | n.rf.s. | yes |
| 37 |  | Blantyre | storage room | 50 | < 30 °C | - | - | - | 25.9 | n.rf.s. | yes |
| 38 |  | Blantyre | - | - |  | - | - | - | n.rt.s. | n.rf.s. | no |
| 39 |  | Blantyre | - | - |  | - | - | - | n.rt.s. | n.rf.s. | no |
| 40 |  | Blantyre | - | - |  | - | - | - | n.rt.s. | n.rf.s. | no |
| 41 |  | Blantyre | - | - |  | - | - | - | n.rt.s. | n.rf.s. | no |
| 42 | drug store | Chikwawa | - | - |  | - | - | At drug stores,  samples were collected by mystery shopper approach,  therefor no information on actual storage conditions,  no temperature loggers placed,  and no questionnaires completed | | | |
| 43 |  | Chikwawa | - | - |  | - | - |  |  |  |  |
| 44 |  | Chikwawa | - | - |  | - | - |  |  |  |  |
| 45 |  | Chikwawa | - | - |  | - | - |  |  |  |  |
| 46 |  | Chikwawa | - | - |  | - | - |  |  |  |  |
| 47 |  | Chikwawa | - | - |  | - | - |  |  |  |  |
| 48 |  | Neno | - | - |  | - | - |  |  |  |  |
| 49 |  | Ntcheu | - | - |  | - | - |  |  |  |  |
| 50 | wholesaler  & CMST | Blantyre | storage room | - |  | 30 | < 25 °C | At wholesalers,  samples were collected by mystery shopper approach,  therefor no information on actual storage conditions,  no temperature loggers placed,  and no questionnaires completed | | | |
| 51 |  | Blantyre | storage room | - |  | 30 | < 30 °C |  |  |  |  |
| 52 |  | Blantyre | storage room | 60  570^e^ | < 30 °C  < 30 °C | 30  100^e^ | 2-8 °C  2-8 °C |  |  |  |  |
| 53 |  | Blantyre  (CMST) | storage room | 400^e^  300^e^ | < 25 °C  < 30 °C | 80^e^  80^e^  100^e^ | 2-8 °C  < 25 °C  < 25 °C |  |  |  |  |
| 54 |  | Blantyre | storage room | - |  | 50 | 2-8 °C |  |  |  |  |
| 55 |  | Blantyre | storage room | 60 | < 30 °C | 100^e^ | < 25 °C |  |  |  |  |
| 56 |  | Lilongwe | storage room | - |  | 30  100^e^  100^e^ | 2-8 °C  2-8 °C  < 30 °C |  |  |  |  |
| 57 |  | Lilongwe | storage room | 300^e^ | < 25 °C | - |  |  |  |  |  |
| 58 |  | Blantyre | - | - |  | - |  |  |  |  |  |
| 59 |  | Blantyre | - | - |  | - |  |  |  |  |  |
| 60 |  | Blantyre | - | - |  | - |  |  |  |  |  |
| 61 |  | Blantyre | - | - |  | - |  |  |  |  |  |
| 62 |  | Blantyre | - | - |  | - |  |  |  |  |  |

*Additional File 1: List of included health facilities, pharmacies and wholesalers, with sizes of collected misoprostol and oxytocin samples, and storage conditions. The cases where actual storage conditions are in conflict with storage conditions stated on the label are highlighted by exclamation marks.*

*RT = room temperature. n.rf.s. = no refrigerated storage of oxytocics; n.rt.s. = no room temperature storage of oxytocics. CMST = Central Medical Stores Trust.*

*^a^ temperature logger placed at misoprostol storage place; ^b^ temperature logger placed at oxytocin storage place (if different)*

*^c^ according to information by health facility personnel, logger has not been kept consistently in refrigerator, therefore excluded from further calculations.*

*^d^ recorded temperature indicates storage of temperature logger in refrigerator for at least part of the time, therefore excluded from further calculations. ^e^ higher number of tablets / vials purchased as replacement samples / for additional stability testing.*
