# Supplementary material for: Quality, availability and storage conditions of oxytocin and misoprostol in Malawi
Source: BMC Pregnancy Childbirth. 2020 Mar 29;20:184. doi: 10.1186/s12884-020-2810-9 (PMC7104524; doi:10.1186/s12884-020-2810-9)
Supplement: Supplementary file 2 — Additional file 2. Questionnaire and consent form. [file 12884_2020_2810_MOESM2_ESM.pdf]

Appendix 7 Quality, Availability and Knowledge of Rational Use and Storage Requirements of Oxytocics in Malawi

SAMPLE SITE & DRUG PURCHASE RECORD

Name of survey site: ______________________ Date of visit: __________

Contact in case of follow-up questions: Name: ______________ Mobile number: __________

Description of location (pharmacy /maternity ward): ______________ CHAM facility? YES □ NO □ Functioning fridge available? YES □ NO □

Photo taken: YES □ NO □ measured room temperature at facility (+time of measurement): __________

Temperature loggers placed? YES □ NO □ Serial number and location of placed temperature loggers: __________

**Drug samples collected**	Desired quantity	Obtained quantity	QOM reference number	Reason for not obtaining the desired quantity: - not available at all (NA) - out of stock (OOS) - not available in sufficient amount (NASA) - other (please describe!)	Price per tabs/vial in MWK (if applicable)	Stock on hand (in tabs/vials)	Monthly consumption (in tabs/vials, based on last six months)	Stockout time in last 6 months (= total number of days when this medicine was not available)	SOP for oxytocic storage available? (yes/no) If yes, please take picture / copy	STGs for oxytocics available? (yes/no) If yes, please take picture / copy	Storage conditions: 1) refrigerated (r) /not refrigerated (nr) 2) in original package (op)/out of original package (nop) 3) protected from light (pr) /not protected from light (npr)	1) Thermometer kept with oxytocic medicines (yes/no) 2) Temperature recorded daily (yes/no) If yes, please take picture
						If possible, please take picture / copy of stock book!						
Misoprostol 0,2mg tab.	50										(r) (nr) (op) (nop) (pr) (npr)	1) yes no 2) yes no
Oxytocin 10 IU vial	10										(r) (nr) (op) (nop) (pr) (npr)	1) yes no 2) yes no

Arrival dates and origin (eg CMST, donation) of last two orders: __________

Expected arrival date and origin of next order: __________

What do you do with expired samples? __________

Comments: __________

If samples taken from bulk / without original package, please take picture of original package / bulk container (showing label, batch number, expiry date, manufacturing date, name / adress of manufacturer)!

Collected samples were replaced: YES □ NO □ Collected samples were paid for: YES □ (Attach receipt!) NO □

Name of sampling person: __________ Signature: __________

Name of accompanying person: __________ Signature: __________

Name of person responsible for health facility: __________ Signature: __________

Appendix 8 Quality, Availability and Knowledge of Rational Use and Storage Requirements of Oxytocics in Malawi

Name of survey site: ______________________ Date of visit: ____________

QUESTIONNAIRE FOR KNOWLEDGE OF RATIONAL USE AND STORAGE REQUIREMENTS OF OXYTOCICS:

1. Profession / training level of person responsible of oxytocics /who administers oxytocics: □ Nurse □ Pharmaceutical technician □ Pharmacist
   □ other: ______________________
2. How long has he/she been doing this work? □ less than 1 year □ 1-3 years □ 4-7 years □ 7-12 years □ more than 12 years
3. Has he/she ever attended training on storage, distribution and handling procedures of cold chain medicines? □ yes □ no
   If yes, how many times has he/she attended such a course within the last three years? □ once □ twice □ thrice □ more than thrice □none within last three years
4. How should oxytocin be stored? □ depending on manufacturer □ at room temperature □ in a fridge
5. How should misoprostol be stored? Multiple answers possible! □ at a dry place □ at room temperature □ in a fridge □ in aluminium blisters

Oxytocin, that is used right now (manufacturer, declared storage conditions): ______________________

Other oxytocin products (+ its storage conditions) that have been used in the last 12 months: ______________________

Misoprostol, that is used right now (manufacturer, declared storage conditions): ______________________

Other Misoprostol products that have been used in the last 12 months: ______________________

6. Have you ever experienced ineffective oxytocics? Misoprostol: □ yes □ no Oxytocin: □ yes □ no
   If yes, what brand? ______________________
   What actions have been taken? Multiple answers possible!
   □ notify authorities (DHO/PMPB) □ notifiy supplier (CMST, Wholesaler) □ nothing □ buy different brand
7. Do you usually also have (methyl-)ergometrine on stock? □ yes □ no
8. What time do you switch off the fridge in the facility? □ evening □ over the weekend □ never switched off

9. How often do you have power black-outs (approx.)? □ less than once a month □ 1-3 times a month □ once a week □ 1-3 times a week □ daily

10. How do you maintain appropriate storage condition in the event of power failure? □ gas □ solar □ no measures

11. Do you have an automated functional generator system in case of power failure? □ yes □no

For maternity wards / health centers / health posts only:

Number of deliveries in last 6 months:________________ Number of reported cases of PPH in last 6 months:________________

1. Have the numbers of deliveries in the last 6 months significantly increased or decreased? □ yes (increased: □ decreased: □) □ no
   If yes, reasons? Multiple answers possible!
   □ availability of infrastructure (e.g. power / water)
   □ availability of medical staff
   □ availability of medicines / medical devices
   □ lack of family planning
   □other:________________

2. When do you give oxytocin to prevent / treat PPH? □ a) always after delivery of child □ b) always before delivery of child □ c) only when woman is bleeding

3. When do you give misoprostol to prevent / treat PPH? multiple answers possible!
   □ no misoprostol on stock □ if oxytocin is not available □ if oxytocin is not working □ if home delivery is planned/most likely
   □ other:________________

PARTICIPANT INFORMATION LEAFLET AND CONSENT FORM (COPY FOR INVESTIGATOR)

Title of the research project: A survey on quality, availability and knowledge of rational use and storage requirements of oxytocics in Malawi.

Principal investigator:
Felix Khuluza, Pharmacy Department, College of Medicine, University of Malawi
Private Bag 360, Chichiri, Blantyre 3, Malawi
Tel. Secretariat +265 (0) 187 4107; www.medcol.mw
Mobile: +265 (0)999 289874
Email: fkhuluza@medcol.mw or fkhuluza@cartafrica.org

Nhomsai Hagen, Pharmaceutical Institute, University of Tuebingen, Auf der Morgenstelle 8, 72076 Tuebingen, Germany; Tel. Secretariat: +49 (0)7071 2972460; www.uni-tuebingen.de
Email: Nhomsai.hagen@uni-tuebingen.de

You are being invited to take part in the survey as titled above. Please take some time to read the information presented here, which will explain the details of this survey. Please ask the study staff any questions about any part of this project that you do not fully understand. It is very important that you are fully satisfied that you clearly understand what this research entails and how you could be involved. Also, your participation is **entirely voluntary** and you are free to decline to participate. If you say no, this will not affect you negatively in any way whatsoever. You are also free to withdraw from the study at any point, even if you do agree to take part.

This study has been approved by the College of Medicine Research and Ethics Committee and will be conducted according to the ethical guidelines and principles of the international Declaration of Helsinki, Malawi Guidelines for Good Clinical Practice.

What is this research study all about?

- *This study will be conducted in various districts in Malawi*
- *The aim of the study is to investigate quality, availability and knowledge of rational use and storage requirements of oxytocics at different points of care and different points of the supply chain in Malawi*

Why have you been invited to participate?

- *You have been asked to participate in this study because you are involved in distribution and / or administration of oxytocics and your knowledge and experience in this area will help in collecting vital information for this study.*

What will your responsibilities be?

- *Your responsibility is to provide samples of oxytocics and all information that you know on the questions asked, as honestly and as openly as you can.*

- *To investigate storage conditions of oxytocics, we will place single-use temperature loggers where you store oxytocin and misoprostol, which will be re-collected after three months. Please ensure, that these loggers are not moved during these three months.*

Will you benefit from taking part in this research?

- *The benefits of participating in this study is that the data will help policy makers on possible ways of improving quality and availability of oxytocics in the country as such you will not directly benefit.*

Are there any risks involved in your taking part in this research?

- *There are no risks in taking part in this research and all participants' names and places will be kept confidential, nor will they be published.*

If you do not agree to take part, what alternatives do you have?

- *You are free to decline to take part in this study. Nothing will happen to you if you decide to decline. You can also decide to decline parts of the study (e.g. placing temperature loggers)*

Who will have access to the records of the data?

- *The data will be kept confidential. Only the investigator in this study will have access to the data. When the data is published, we will not use names or any other information that may lead to readers identifying you.*
- *Results will be presented to you via a letter to your DHO, before being made available to any other party or to the public or being published in reputable journals.*

Will you be paid to take part in this study and are there any costs involved?

- *No you will not be paid to take part in the study. There will be no costs involved for you, if you do take part. The samples you provide will be replaced or paid for.*

Is there anything else that you should know or do?

- *You can contact PI at tel: 0999289874 if you have any further queries or encounter any problems.*
- *You can contact the Secretariat of College of Medicine Research and Ethics Committee at* 0111871 911 *if you have any concerns or complaints that have not been adequately addressed by the study staff.*
- *You will receive a copy of this information and consent form for your own records.*

Declaration by participant

By signing below, I .... agree to take part in a research study entitled *A Survey on quality, availability and knowledge of rational use and storage requirements of oxytocics in Malawi.*

I declare that:

- I have read or had read to me this information and consent form and it is written in a language with which I am fluent and comfortable.
- I have had a chance to ask questions and all my questions have been adequately answered.
- I understand that taking part in this study is **voluntary** and I have not been pressurized to take part.
- I may choose to leave the study at any time and will not be penalized or prejudiced in any way.

Signed at (*place*) .... on (*date*) .... 2018.

....

Signature of participant

Declaration by investigator

I *(name)* .... declare that:

- I explained the information in this document to ....
- I encouraged him/her to ask questions and took adequate time to answer them.
- I am satisfied that he/she adequately understands all aspects of the research, as discussed above and that his/her participation is voluntary
- I did not use an interpreter.

Signed at (*place*) .... on (*date*) .... 2018.

....

Signature of investigator

Further queries should be addressed to:

The Chairperson
College of Medicine Research and Ethics Committee (COMREC)
Private Bag 360, Chichiri, Blantyre 3
Tel: + 265 (0) 1871 911
