# Supplementary material for: Quality, availability and storage conditions of oxytocin and misoprostol in Malawi
Source: BMC Pregnancy Childbirth. 2020 Mar 29;20:184. doi: 10.1186/s12884-020-2810-9 (PMC7104524; doi:10.1186/s12884-020-2810-9)
Supplement: Supplementary file 3 — Additional file 3. Results from Interviews. [file 12884_2020_2810_MOESM3_ESM.docx]

**Additional File 3: Results from Interviews**

**1) Stockout times of oxytocin and misoprostol at health facilities in the last 6 months**


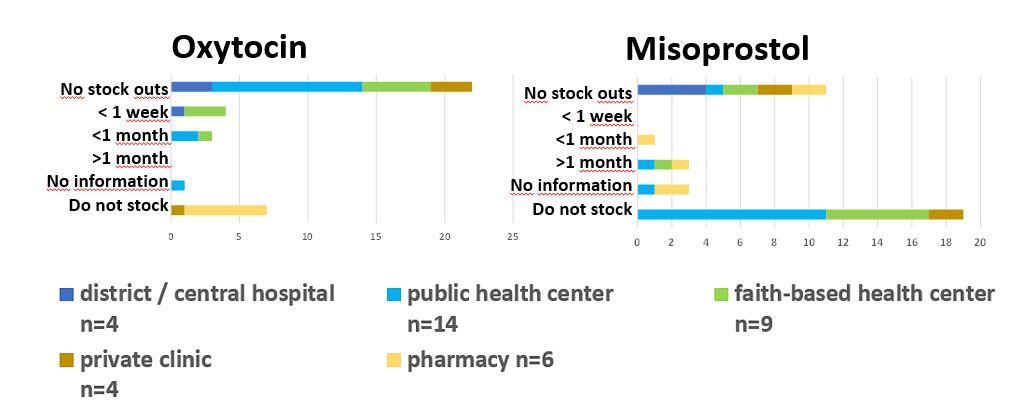


In case of public and faith-based health facilities and private clinics, this information was verified by inspecting the stock cards for the respective medicines. n= number of facilities.

**2) Availability of Standard Operating Procedures (SOPs) for (oxytocic) storage**


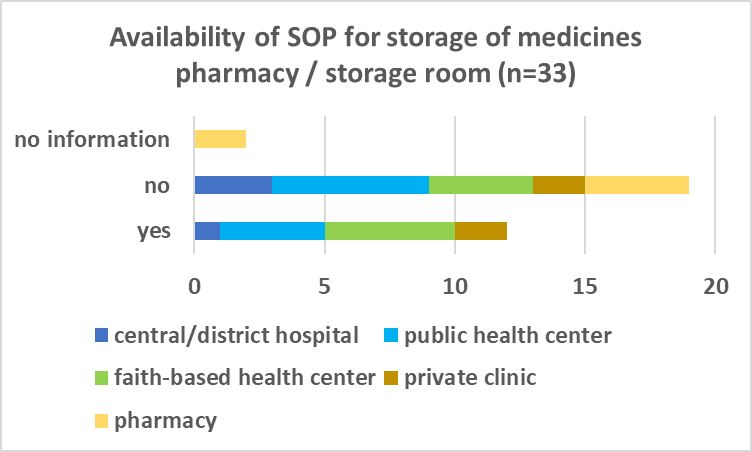


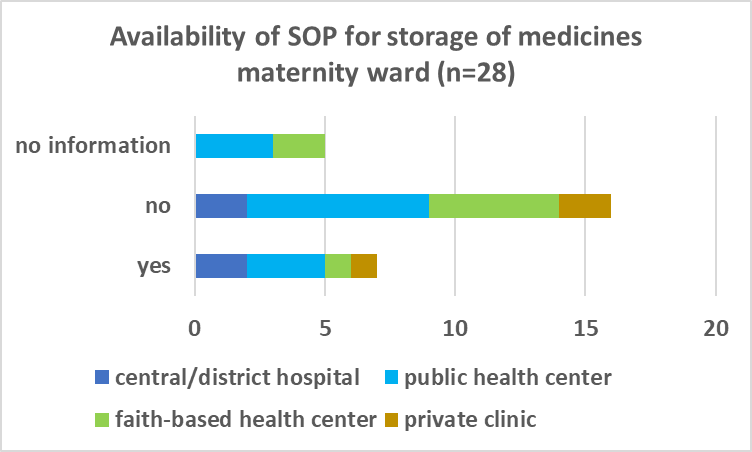


Specific SOPs just for the storage of oxytocin or misoprostol were not available at any health facility /pharmacy.

**3) Profession / training level of person responsible for oxytocics / who administers oxytocics**


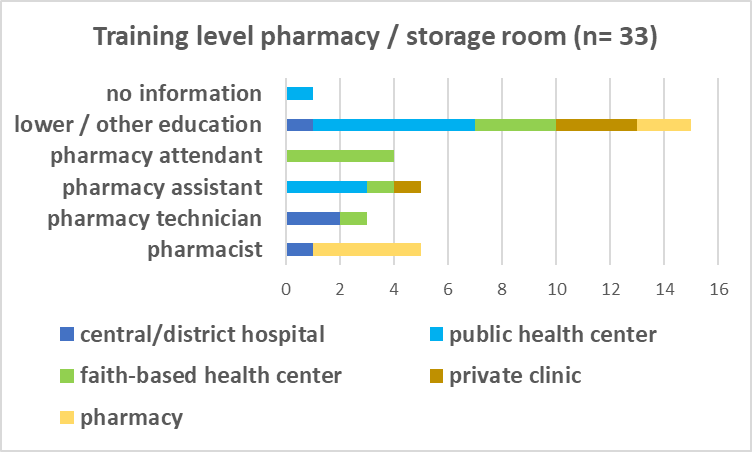


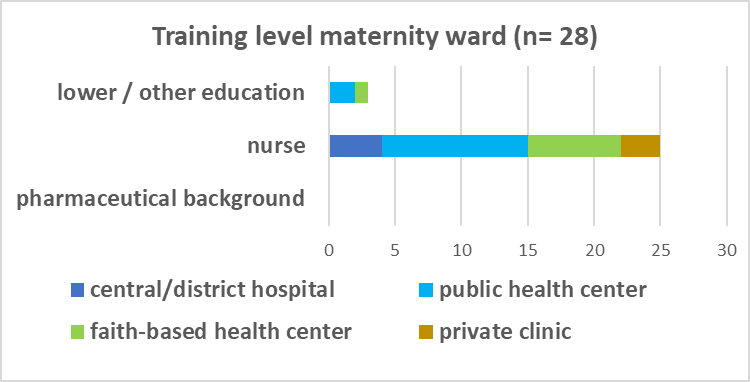


**4) Work experience of person responsible for oxytocics / who administers oxytocics**


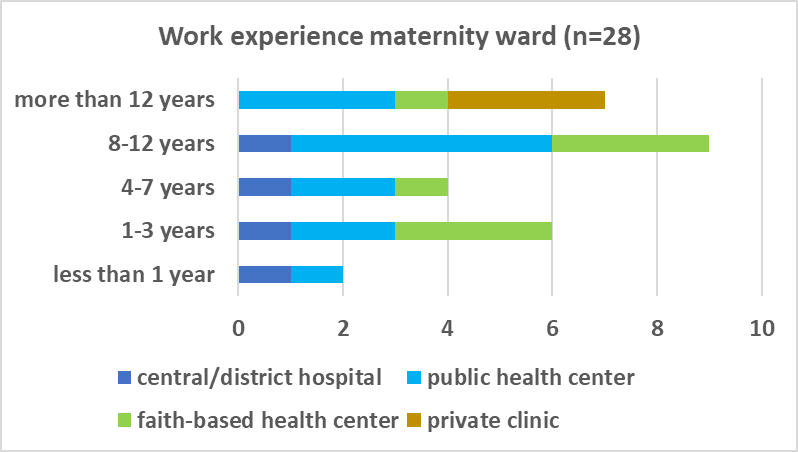


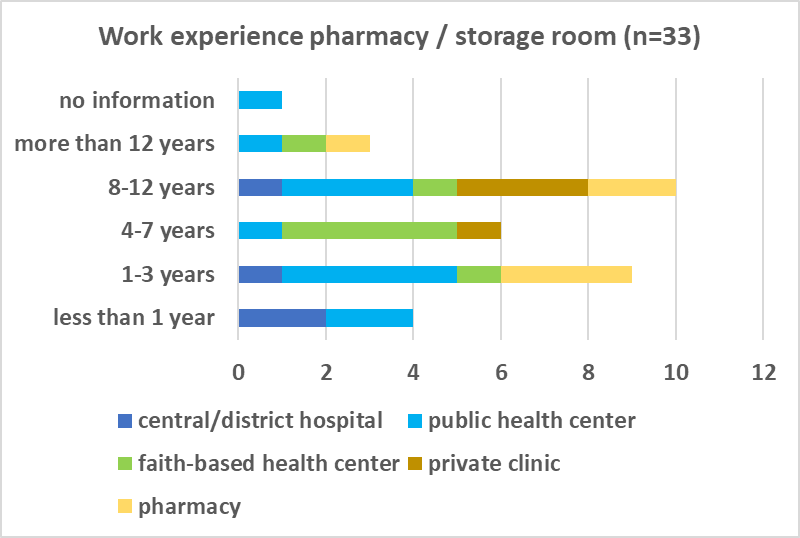


**5) Storage of misoprostol**


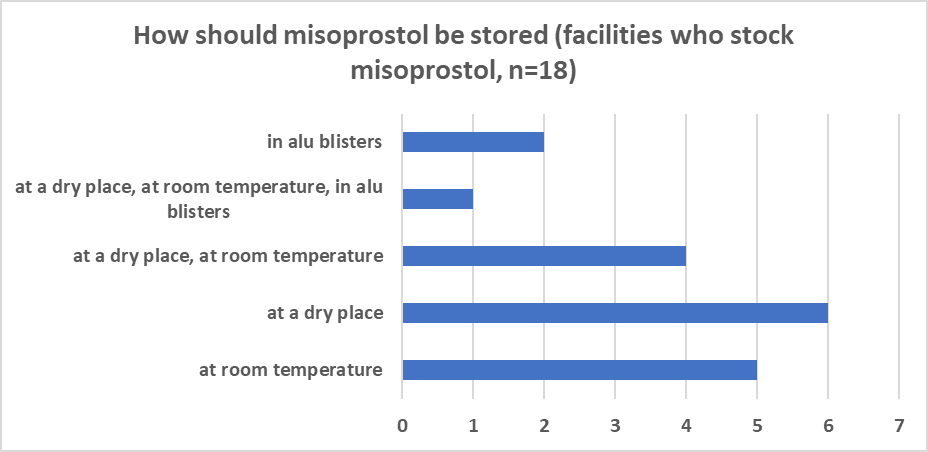


Question as multiple choice. The importance of aluminium /aluminium blisters as primary packaging is not well known (only 3 out of 18 ticked “in aluminium / aluminium blisters”); n= 18 (12 health facilites + 6 pharmacies)

**6) At what time do you switch off the fridge in the facility?**

One health worker from a public health center ticked “in the evening” on the question “at what time do you switch of the fridge in the facility?”. One health worker from a faith-based health center ticked “over the weekend” when asked this question. All other health worker ticked “never switched off”.

**7) When do you give misoprostol do prevent / treat PPH? Multiple answers possible**


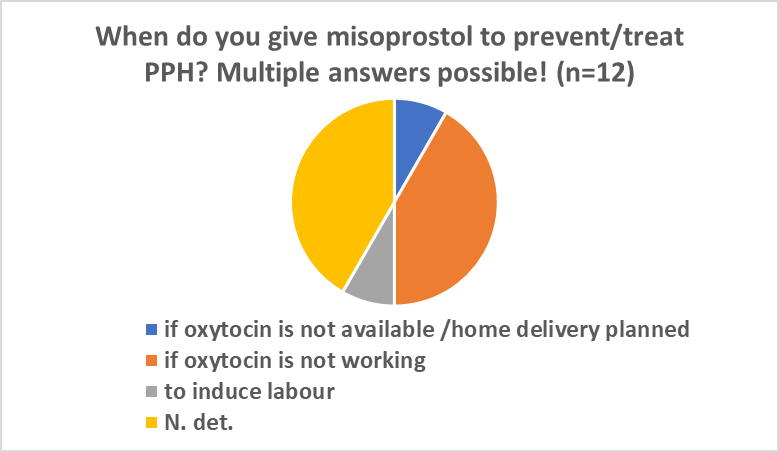


Answers of all 12 health facilities, who stock misoprostol. N.det.: not determined.

**8) Numbers of deliveries at health facilities, and PPH rates**

| **Type of facility** | **Facility number** | **number of deliveries in the last 6 months** | **% reported PPH/deliveries last 6 months** | **have N° of deliveries increased /decreased?** |
| --- | --- | --- | --- | --- |
| central hospital | 1 | 6768 | N.det. | decreased |
| district hospital | 2 | 2130 | 3.38 | NA (person new at facility) |
|  | 3 | 780 | 1.58 | increased |
|  | 4 | 3847 | 0.94 | increased |
| public health center | 5 | 260 | 0.38 | N.det. |
|  | 6 | N.det. | N.det. | N.det. |
|  | 7 | 1455 | 1.24 | increased |
|  | 8 | 1680 | 0.36 | increased |
|  | 9 | 520 | N.det. | increased |
|  | 10 | 203 | 0.99 | increased |
|  | 11 | 30 | 33.3 | varies with months |
|  | 12 | 318 | 3.1 | increased |
|  | 13 | 141 | 0 | N.det. |
|  | 14 | 620 | N.det. | increased |
|  | 15 | 330 | 3.3 | increased |
|  | 16 | 540 | 1.1 | N.det. |
|  | 17 | 59 | 3.39 | varies with months |
|  | 18 | N.det. | N.det. | N.det. |
| faith-based health center | 19 | N.det. | N.det. | N.det. |
|  | 20 | 1293 | 1.47 | increased |
|  | 21 | 1800 | 1.3 | increased |
|  | 22 | 78 | 3.85 | decreased |
|  | 23 | 220 | 0.9 | decreased |
|  | 24 | 290 | 6.21 | decreased |
|  | 25 | 23 | 0 | increased |
|  | 26 | 32 | 6.25 | increased |
|  | 27 | 241 | 0.83 | increased |
| private clinic | 28 | 15 | 0 | neither |
|  | 29 | 7 | 0 | decreased |
|  | 30 | 92 | 0 | N.det. |
|  | 31 | no delivery services | | |

N.det: not determined. PPH: Post-partum haemorrhage

As reasons for **increased** numbers of deliveries were mentioned:

- Lack of familiy planning (n=8)
- Increased teenage pregnancies (n=2)
- People prefer delivering in health center rather than at home (n=3)
- Closing of nearby health center (n=1)

As reasons for **decreased** numbers of deliveries were mentioned:

- Successful family planning campaign (n=2)
- Women are referred to district hospitals due to birth complications (n=1)
- Expensive, people prefer public health center (n=1, private clinic)
- NA (n=1)
