# Supplementary material for: Quality, availability and storage conditions of oxytocin and misoprostol in Malawi
Source: BMC Pregnancy Childbirth. 2020 Mar 29;20:184. doi: 10.1186/s12884-020-2810-9 (PMC7104524; doi:10.1186/s12884-020-2810-9)
Supplement: Supplementary file 4 — Additional file 4. Related substances chromatogram of Misoprostol Tablets 200mcg by swiss parenterals Pvt. Ltd. [file 12884_2020_2810_MOESM4_ESM.docx]

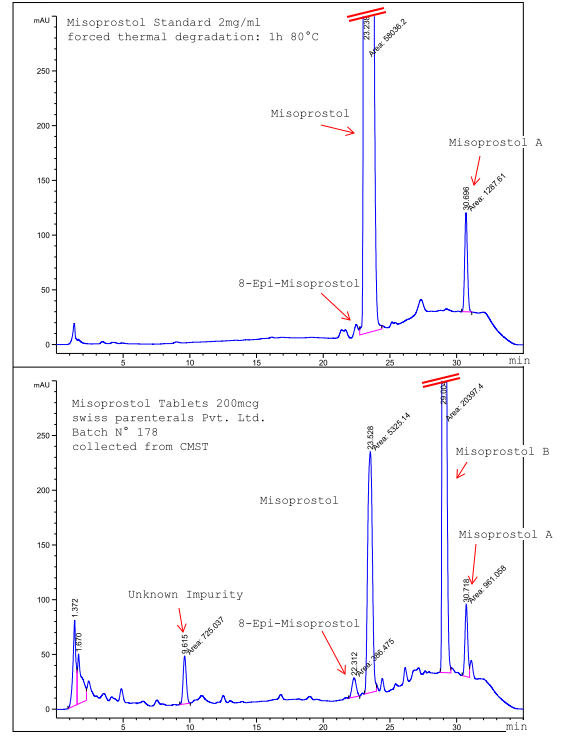


***Additional File 4: Related substances chromatogram of Misoprostol Tablets 200mcg by swiss parenterals Pvt. Ltd.*** *Chromatographic conditions according to Kahsay et al. 2015 (Ref. 28):
HPLC: Agilent Infinity 1260 II
Column: Dr. Maisch ReproSil-XR 120 C18, 5µm, 150mm x 4,6 mm with guard
Mobile phase A: ACN**-H_2_0-MeOH, 28:69:3 (v/v/v); mobile phase B: ACN-H_2_0-MeOH, 47:50:3 (v/v/v)
Gradient (time [min]/%B): 0/0, 5/0 to 15/35, 20/35 to 25/95, 30/95 to 32/0, 35/0
Flow rate: 1.5ml/min; UV detection: 200nm
Column oven: 35°C. Samples in refrigerated autosampler at 4°C
Injection volumes: 200 µl (sample, 0.4mg/ml), 50 µl (reference, 2mg/ml)*

*Misoprostol Ph.Eur. reference standard (batch N° 3.0) was dissolved in ACN-H_2_0 45:55 (v/v) at a concentration of 2mg/ml. For forced thermal degradation, this solution was placed for 1h in an oven at 80 °C.*

*Sample solution was prepared as described in Kahsay et. al., using 3 tablets per sample.*
